# Advantages of a second ventilation circuit when using a double-lumen endotracheal tube

**DOI:** 10.1007/s12630-019-01553-y

**Published:** 2019-12-17

**Authors:** Gerard Bruin

**Affiliations:** Trillium Health Partners, Credit Valley Site, Mississauga, ON Canada

**To the Editor,**

In thoracic surgery, lung isolation is frequently achieved using a double-lumen endotracheal tube (DLT). Outward migration of the DLT away from the carina and loss of lung isolation can occur despite adequate initial positioning of the tube using a bronchoscope. The use of a second ventilation circuit attached to the operative lumen of the DLT is a useful method to detect a minor DLT malposition (and leaks) while it can still be easily corrected (Figure). The following describes the second circuit technique that we use to detect early outward migration of the DLT.

**Figure:**
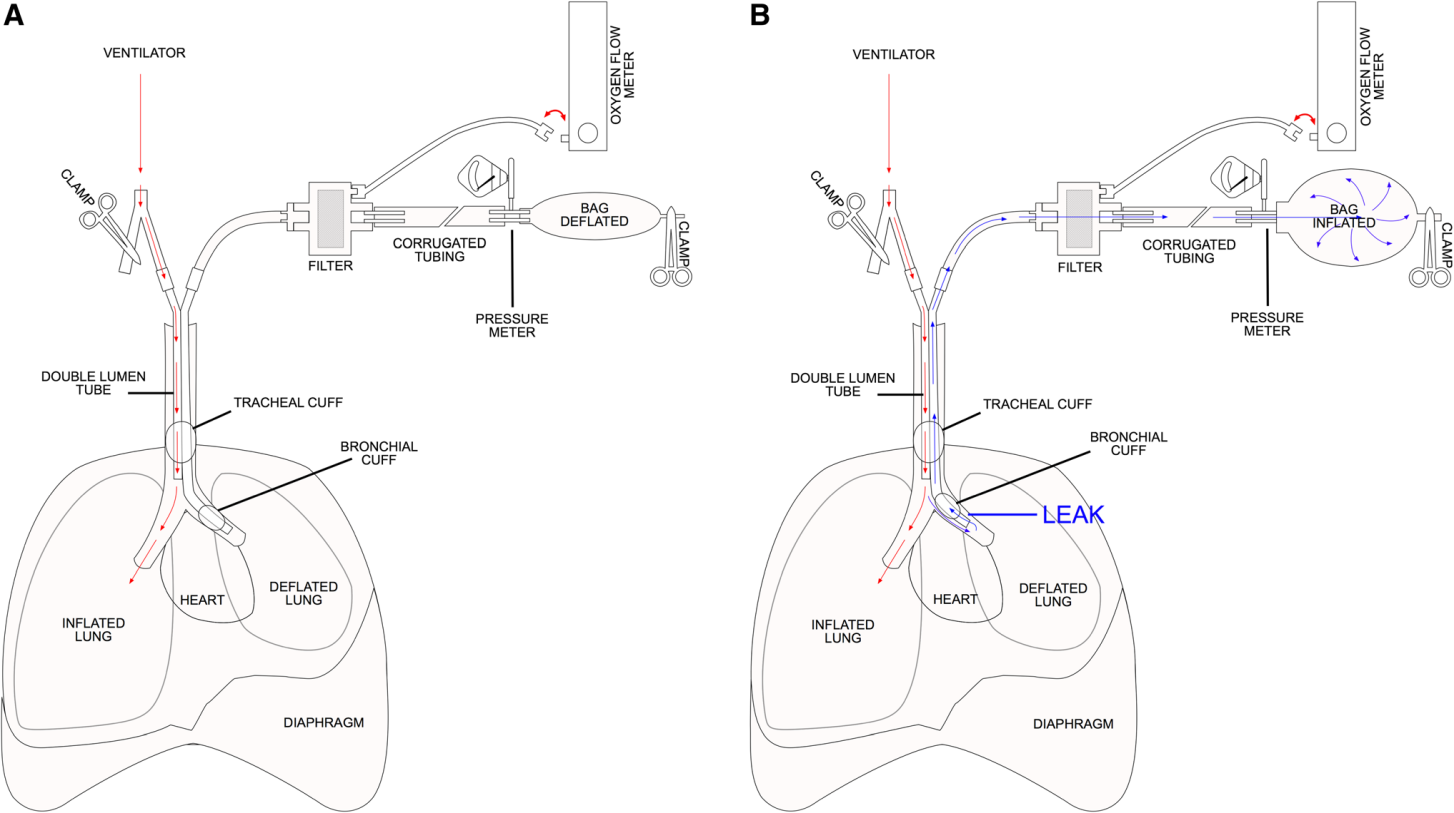
A) The use of a second circuit during lung isolation for thoracic surgery. A double-lumen endotracheal tube (DLT) is in the correct position with the endobronchial cuff secure within the bronchus. The tubing of the second circuit is disconnected from the auxiliary flow meter when the circuit is used for monitoring. The clamp is removed before connecting the circuit to the auxiliary flow meter when ventilating with the circuit. B) The DLT has migrated outward and the endobronchial cuff has begun to migrate into larger lumen carina area resulting in a leak around the cuff. Air from the ventilated lung goes around the cuff and exits out the lumen of the DLT and begins to fill the ventilation bag of the second circuit.

We start one-lung ventilation (OLV) and attach the second circuit immediately after the patient is intubated and the DLT is confirmed to be in correct position (Figure, panel A). Next, watching the bag in the circuit, the minimal amount of air required to prevent a leak around the endobronchial cuff is determined (eVideo available as Electronic Supplementary Material [ESM] at https://vimeo.com/370217402; time stamp: 5 min 26 sec). Too little air in the cuff during positive pressure ventilation will result in a leak of air from the ventilated lung around the cuff and into the operative lung thus filling the ventilation bag of the second circuit. Importantly, if no air is required in the cuff (e.g., because an over-sized DLT being used), this method to detect minor malposition of the DLT will not work.

With migration of the DLT outward from the carina, the endobronchial cuff will begin to move into the larger lumen carinal area resulting in a leak around the cuff. This will also fill the bag of the second circuit (Figure, panel B). This prompts us to check and reposition the DLT prior to complete loss of lung isolation. The leak will not inflate the lung on the side of surgery.

We also monitor the DLT position during subsequent patient positioning because we have the patient on OLV (ESM eVideo time stamp: 6 min 15 sec). Furthermore, while on OLV and before chest entry, we intermittently ventilate and flush the circuit with oxygen. To ventilate with the circuit, we remove the clamp over the hole in end of the bag before connecting the circuit to the auxiliary oxygen flow meter. While ventilating with the circuit, we control pressure in the circuit by pinching off the hole in the bag between the thumb and index finger.

With the patient on OLV prior to chest entry, we often see the bag inflate and deflate in response to movement of the non-operative (ventilated) lung. This is not a leak as there is no net accumulation of air in the bag. With use of the circuit, this indirect ventilation to the surgical lung is with 100% oxygen from the reservoir of our circuit. This helps facilitate deflation of the lung via absorption atelectasis (ESM eVideo time stamp: 9 min 36 sec).

The second circuit allows slower and more controlled inflations of the surgical lung when re-expansion is required.^1^ We can position the bag and manometer to best observe the surgical field. If we need to release pressure quickly, we open the hole in the bag by opening our hand (ESM eVideo time stamp: 10 min 51 sec).

In summary, using a second circuit can allow for the monitoring of early DLT malposition and thus warn of impending loss of lung isolation. By indirectly ventilating the lung with 100% oxygen while on OLV prior to chest entry we achieve superior subsequent lung deflation. We also have better control of lung inflation to test for leaks and assist with lung recruitment in the operative lung after the lung resection has been completed.

## Electronic supplementary material

Below is the link to the electronic supplementary material.

Supplementary material 1 (MP4 39885 kb)

Supplementary material 1 (PDF 63 kb)
