# Supplementary material for: Advantages of a second ventilation circuit when using a double-lumen endotracheal tube
Source: Can J Anaesth. 2019 Dec 17;67(6):766–7. doi: 10.1007/s12630-019-01553-y (PMC7214381; doi:10.1007/s12630-019-01553-y)
Supplement: Supplementary file 2 — Supplementary material 1 (PDF 63 kb) [file 12630_2019_1553_MOESM2_ESM.pdf]

eVideo legend

Video demonstrates the use of a second ventilating circuit attached to the operative lumen of a double-lumen endotracheal tube during one-lung ventilation for thoracic surgery in order to help detect leaks and endotracheal tube malposition. The video can be seen at https://vimeo.com/370217402

Time location	Topic
1 min 17 sec	The components of the circuit that are assembled from CPAP circuit parts.
3 min 7 sec	The DLT will move outward after correct placement.
5 min 26 sec	The correct amount of air in the endobronchial cuff is just enough to stop the leak.
5 min 51 sec	The second circuit bag when the DLT is moved outward and the endobronchial cuff begins to move into the carina.
6 min 15 sec	Using the circuit to monitor the DLT position during transitioning to lateral decubitus.
9 min 36 sec	The bag movement prior to chest opening where indirect ventilation with 100% oxygen of the operative lung can be seen.
10 min 10 sec	The difference in how we use the circuit when we use it for monitoring and when it is used for ventilating.
10 min 51 sec	Monitoring the pressure in the circuit and how we ventilate and inflate the lung for testing against leaks along the staple line and recruit atelectatic lung during re-expansion.

CPAP = continuous positive airway pressure; DLT = double-lumen endotracheal tube.
